# Supplementary material for: Autonomous robotic nanofabrication with reinforcement learning
Source: Sci Adv. 2020 Sep 2;6(36):eabb6987. doi: 10.1126/sciadv.abb6987 (PMC7467688; doi:10.1126/sciadv.abb6987)
Supplement: abb6987_SM.pdf [file abb6987_SM.pdf]

Science Advances
AAAS

advances.sciencemag.org/cgi/content/full/6/36/eabb6987/DC1

Supplementary Materials for

Autonomous robotic nanofabrication with reinforcement learning

Philipp Leinen, Malte Esders, Kristof T. Schütt, Christian Wagner*, Klaus-Robert Müller*, F. Stefan Tautz

*Corresponding author. Email: c.wagner@fz-juelich.de (C.W.); klaus-robert.mueller@tu-berlin.de (K.-R.M.)

Published 2 September 2020, *Sci. Adv.* **6**, eabb6987 (2020)
DOI: 10.1126/sciadv.abb6987

Other Supplementary Material for this manuscript includes the following:

(available at advances.sciencemag.org/cgi/content/full/6/36/eabb6987/DC1)

Movie S1
